# Role of seizure in neonatal stroke

**DOI:** 10.18632/oncotarget.18212

**Published:** 2017-05-25

**Authors:** Stéphane Auvin, Christiane Charriaut-Marlangue

**Affiliations:** Pediatric Neurology, Robert-Debré University Hospital, APHP & INSERM U1141, Paris, France

**Keywords:** neonatal seizure, stroke, phenobarbital, EEG, rat

Perinatal arterial stroke (PAS) is the most frequent form of cerebral infarction in children with an incidence of 1/2800 to 1/5000 live births. Approximately 60% of newborns with PAS exhibit symptoms, mainly neonatal seizures, while 40% of newborns do not show any symptoms during the neonatal period. In most of case, there is no obvious etiology. PAS seems to occur secondary to a combination of factors that are not fully understood. Currently, there is no specific treatment for neonatal stroke. The current strategy is mainly based on supportive care, including the management of neonatal seizures. However, current available antiepileptic drugs including phenobarbital (PB), the first line treatment, have limited efficacy on neonatal seizures [1].

The initial clinical course of PAS seems favorable without recurrence of infarct but almost all children are left with sequel. The main consequence is the neuromotor impairment (almost 100% of patients) mainly in its unilateral spastic form. Epilepsy is diagnosed in 48% of children by the age of 4-year-old [2]. After the occurrence of neonatal seizures during PAS, there is a nearly 3-fold increased risk of later epilepsy. In these cases, the cumulative incidence of epilepsy by age 10 years is approximately 70% [3]. Neuropsychological consequences are usually observed at school age with a significant decline of IQ in 69% [4].

It is still unclear if a large stroke results in more seizures than a smaller stroke volume with the seizures reflecting the stroke volume or if the seizures contribute to increase the stroke volume by combining the excitotoxicity of the seizure to the anoxo-ischemic insult. The link between seizure burden during the acute phase of PAS and stroke volume is crucial because of the possible treatment consequences. The use of animal model to address this issue allow to control the various parameters such as exact seizure quantification from stroke initiation to exact stroke volume assessment, timing for the treatment and histology study.

In human newborn, seizure suppression is believed to play a role in the long-term prognosis of the underlying cause of the seizure, such as anoxo-ischemic encephalopathy [5]. However, there is no data focusing on the anti-seizure activity and outcome of phenobarbital-treated cases of PAS. Before the design of clinical trials in human newborns, preclinical studies must provide insights on the treatment of neonatal seizures in cases of PAS.

Using a neonatal rat ischemic-reperfusion stroke model, EEG recordings showed two distinctive epileptic events occurring both during ischemia and after reperfusion: bursts of high-amplitude spikes and organized seizures with a pattern close to what is observed in human newborns [6]. The innovative part of the design in our experimental study was the prolonged EEG recording in P7 rats from the ischemic onset to brain samples allowing to study the link between the neonatal brain injury and the seizure burden. Past experimental studies have usually recorded EEG for few hours. We established that both phenobarbital (PB) and levetiracetam decreased the total duration of the bursts of high amplitude spikes. PB also delayed the start of seizures without changing the total duration of epileptic discharges. This observation might have lead to an erroneous conclusion on the efficacy of PB if we would have recorded the animal only for few hours. Both tested drugs in our study did not modify the stroke volume, which suggests that the modification of the quantity of bursts of high amplitude spikes does not influence the infarct size. Indeed, we did not find any correlation between the quantity of burst and the stroke volume. We concluded that the presence of organized seizure was related to the presence of the infarct [6]. Our model represents a good opportunity to evaluate the efficacy of neuroprotective agents on the seizure quantity in order to further delineate the relationship between seizures and stroke volume. It would also provide a good tool to evaluate new anti-seizure compounds for neonatal seizure in PAS refractory to PB.

## References

[1] Donovan (2016). Drugs.

[2] Wanigasinghe (2010). Neurology.

[3] Fox (2016). Neurology.

[4] Westmacott (2009). Stroke.

[5] McBride (2000). Neurology.

[6] Morin (2017). Neurobiol Dis.

